# Controllable *z*-Polarized Spin Current in Artificially Structured Ferromagnetic Oxide with Strong Spin–Orbit Coupling

**DOI:** 10.1021/acs.nanolett.4c05502

**Published:** 2025-01-13

**Authors:** Dongxing Zheng, Jingkai Xu, Qingxiao Wang, Chen Liu, Tao Yang, Aitian Chen, Yan Li, Meng Tang, Maolin Chen, Hanin Algaidi, Chao Jin, Kai Liu, Mathias Kläui, Udo Schwingenschlögl, Xixiang Zhang

**Affiliations:** † Physical Science and Engineering Division, King Abdullah University of Science and Technology (KAUST), Thuwal 23955−6900, Saudi Arabia; ‡ Corelab, King Abdullah University of Science and Technology (KAUST), Thuwal 23955−6900, Saudi Arabia; § Tianjin Key Laboratory of Low Dimensional Materials Physics and Processing Technology, School of Science, Tianjin University, Tianjin 300350, China; ∥ Physics Department, Georgetown University, Washington, D.C. 20057, United States; ⊥ Institute of Physics, Johannes Gutenberg University Mainz, 55099, Mainz, Germany

**Keywords:** Oxide heterostructure, Interfacial reconstruction, Spin−orbit coupling, *z*-spin polarization, magnetization switching

## Abstract

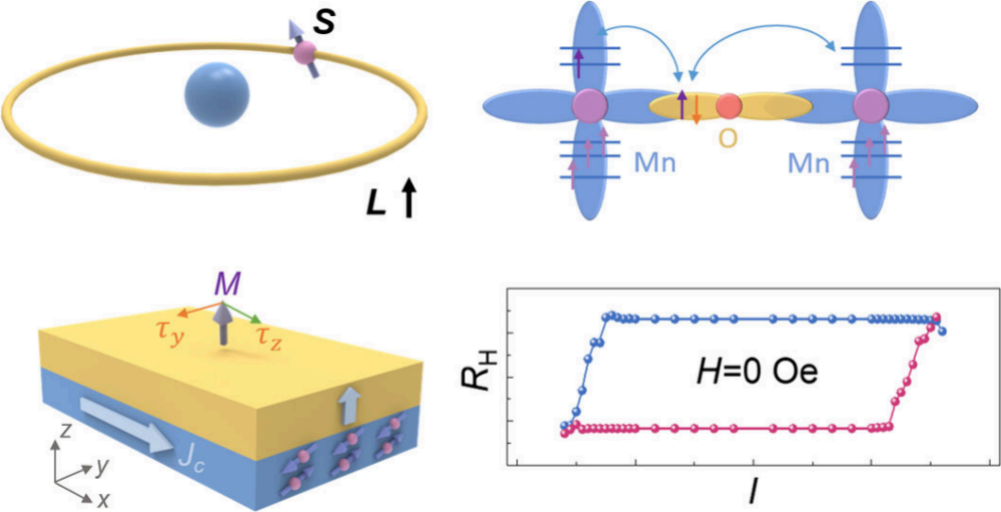

Realizing field-free switching of perpendicular magnetization by spin–orbit torques is crucial for developing advanced magnetic memory and logic devices. However, existing methods often involve complex designs or hybrid approaches, which complicate fabrication and affect device stability and scalability. Here, we propose a novel approach using *z*-polarized spin currents for deterministic switching of perpendicular magnetization through interfacial engineering. We fabricate La_0.67_Sr_0.33_MnO_3_–SrIrO_3_ (LSIMO) thin films with robust spin–orbit coupling and ferromagnetic order through orbital and lattice reconstruction, integrating SrIrO_3_ and La_0.67_Sr_0.33_MnO_3_ materials. Our investigation reveals that *y*- and *z*-polarized spin currents, driven by the spin Hall and spin–orbit precession effects, enable field-free switching of perpendicular magnetization. Notably, the *z*-polarized spin currents are tunable via the in-plane magnetization of LSIMO. These findings present a promising pathway for the development of energy-efficient spintronic devices, offering improved performance and scalability.

Spin–orbit torques (SOTs) offer a promising avenue for realizing low-power, high-speed logic and storage devices by enabling the switching of perpendicular magnetization in materials with strong spin–orbit coupling (SOC).^1−4^ However, traditional SOT mechanisms, particularly those driven by the spin Hall effect with only in-plane spin polarization σ_*y*_, encounter a significant challenge: they cannot reliably realize deterministic switching of a perpendicular magnetization without the assistance of an external magnetic field applied along the current direction (Figure 1a). This reliance on external fields impedes the widespread application of SOT in compact, energy-efficient spintronic devices. To address this limitation and enable field-free perpendicular magnetization switching, various strategies have been explored. These include methods to create an equivalent in-plane magnetic field along the current direction,^5−13^ introduce out-of-plane damping-like torques,^14−27^ or develop hybrid approaches.^28−31^ However, some of these techniques, such as those involving specialized structures or low-symmetry single-crystal nanosheets, pose challenges for industrial scalability.

**Figure 1:**
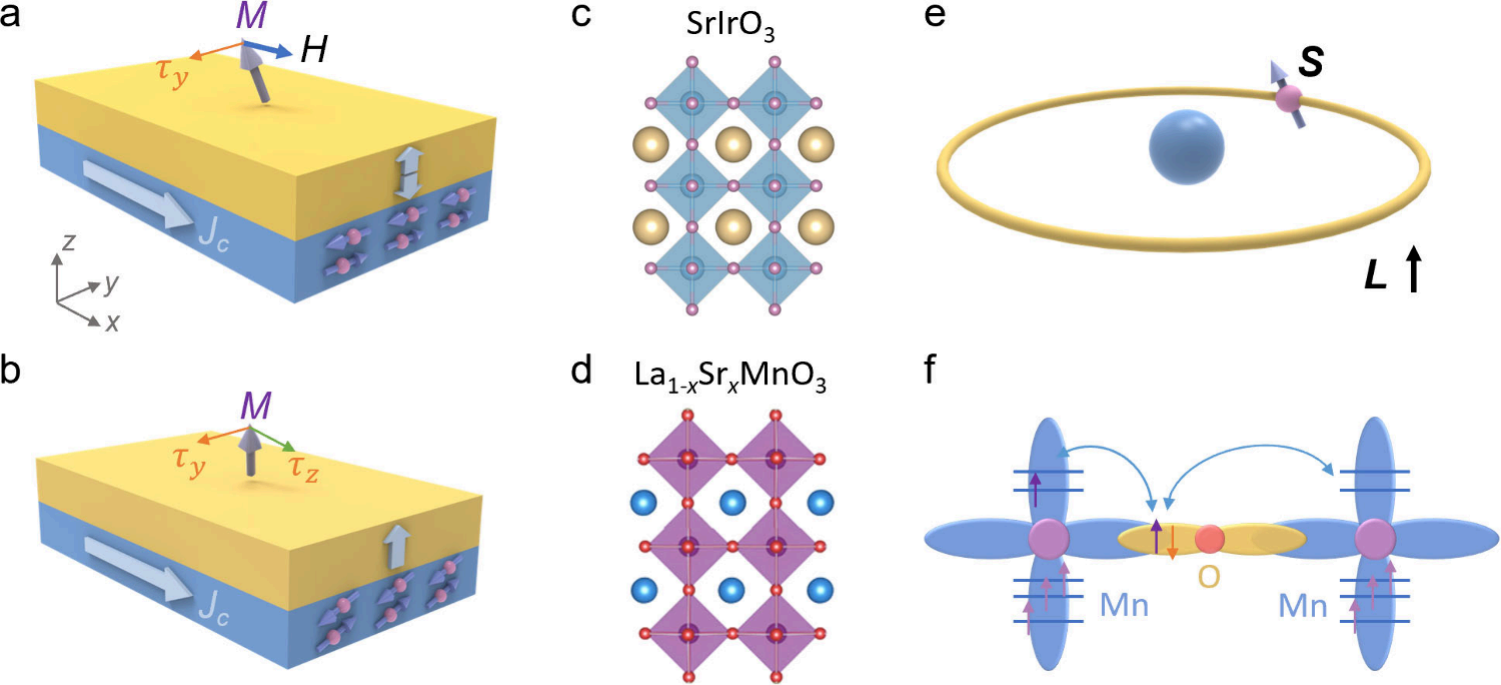
**Schematic illustration of SOT-switching and crystal structure design**. (**a**) Illustration of conventional SOT-switching caused by the spin Hall effect. In this scenario, the perpendicular magnetization layer exhibits the preference for both up and down magnetization states with the same injecting current. Hence, an additional in-plane magnetic field is required to achieve deterministic switching of the perpendicular magnetization. (**b**) Illustration of field-free SOT switching achieved by the combination of both *y*- and *z*-polarized spin current. Schematic crystal structure of (**c**) SrIrO_3_ and (**d**) La_1–*x*_Sr_*x*_MnO_3_. (**e**) Schematic of the SOC effect in SrIrO_3_. (**f**) Schematic of the indirect exchange coupling between Mn ions through an O^2–^ ion.

Recent advancements have demonstrated that SOT with out-of-plane spin polarization σ_*z*_, represented by τ_ADL,*z*_ = *m* × (σ_*z*_ × *m*), holds promise for deterministic magnetization switching with higher efficiency compared to conventional methods.^18−24,32,33^ In this study, we propose a sophisticated design to generate spin current with both in-plane σ_*y*_ and out-of-plane σ_*z*_ spin polarizations to overcome the limitations of nondeterministic switching observed with only σ_*y*_ spin current and realize the deterministic perpendicular magnetization switching (Figure 1b).

Our approach is based on leveraging the unique properties of oxide materials, particularly the lattice and orbital orders reconstruction at artificial oxide interfaces.^34−38^ The multilayers of SrIrO_3_ (SIO) with strong SOC and ferromagnetic La_0.67_Sr_0.33_MnO_3_ (LSMO) are expected to generate spin currents with both in-plane and out-of-plane spin polarization for several reasons. First, both SIO and LSMO are perovskite oxides with closely matching lattice parameters (Figure 1c,d), facilitating the formation of high-quality LSIMO films. Second, due to its strong SOC, SIO exhibits a large spin Hall angle, allowing the fabricated LSIMO films to inherit this property and convert charge current into spin current efficiently (Figure 1e).^39−45^ Third, the magnetic properties of LSMO are governed by the double exchange interaction between the Mn–O–Mn bond. Coupling LSMO with SIO, characterized by strong SOC, is anticipated to enhance the magnetic anisotropies of the LSIMO films (Figure 1f).^35,46−48^ The artificial oxides comprising of LSMO and SIO are expected to generate a tilted-polarized spin current with σ_*y*_ and σ_*z*_ components through the spin Hall effect and spin–orbit precession effect (SOPE).^21,49,50^

In this study, we have successfully realized the field-free switching of perpendicular Co_20_Fe_60_B_20_ (CFB) magnetization by the tilted-polarized spin current generated in an in-plane magnetized artificial oxide [LSMO_0.3_/SIO_0.2_]_120_ (LSIMO) layer; details of the fabrication process are shown in the Method section in Supporting Information. The LSIMO layer is magnetically decoupled from the Mo/CFB/MgO multilayer via a Ti layer. The LSIMO oxide layer was grown by using pulse laser deposition on TiO_2_-terminated SrTiO_3_ substrates. Subsequently, the LSIMO oxide layer was immediately transferred to the Singulus ROTARIS magnetron sputtering system to deposit the Ti(3 nm)/Mo(2 nm)/CFB(0.8 nm)/MgO(2.2 nm)/Ta(2 nm) multilayers. A 2 nm thick Ta layer served as a capping layer to protect the multilayer from oxidation. The current-induced perpendicular magnetization switching can be controlled by changing the in-plane direction of the LSIMO magnetization, consistent with the scenario of the SOPE. Our designs offer new insights into efficient spin current sources that break switching symmetry and facilitate deterministic magnetization switching.

The structure of the LSIMO/Ti/Mo/CFB/MgO/Ta multilayer is schematically shown in Figure 2a. High-quality artificial LSIMO single-crystal films were epitaxially grown on TiO_2_-terminated (001)-oriented SrTiO_3_ (STO) substrates, due to the similar crystal structure and nearly the same lattice constants of SIO, LSMO, and STO (see Figure S1). The high quality of the LSIMO film is confirmed by the appearance of only (00*l*) diffraction peaks in the X-ray θ–2θ diffraction patterns, as shown in Figure 2b. In addition, the clear Laue fringes around the (002) epitaxial peak indicate the flatness and high quality of the film. The flatness of the LSIMO layer is further confirmed by the surface morphology characterization result shown in Figure S3. Figure 2c further illustrates the reciprocal space mapping of the LSIMO and STO reciprocal-lattice points (103). The diffraction point of the LSIMO layer is positioned vertically below the (103) diffraction of the STO substrates, suggesting that the in-plane lattice parameters of the LSIMO layer were fully constrained by the STO substrates. The high-resolution transmission electron microscopy (STEM) image shown in Figure 2d further confirms the excellent crystallinity of the film. The uniform brightness of the La/Sr and Ir/Mn atoms indicates the uniform mixture of LSMO and SIO. Moreover, the uniform growth is further corroborated by the energy-dispersive X-ray spectroscopy (EDXS) mappings in Figure S2.

**Figure 2:**
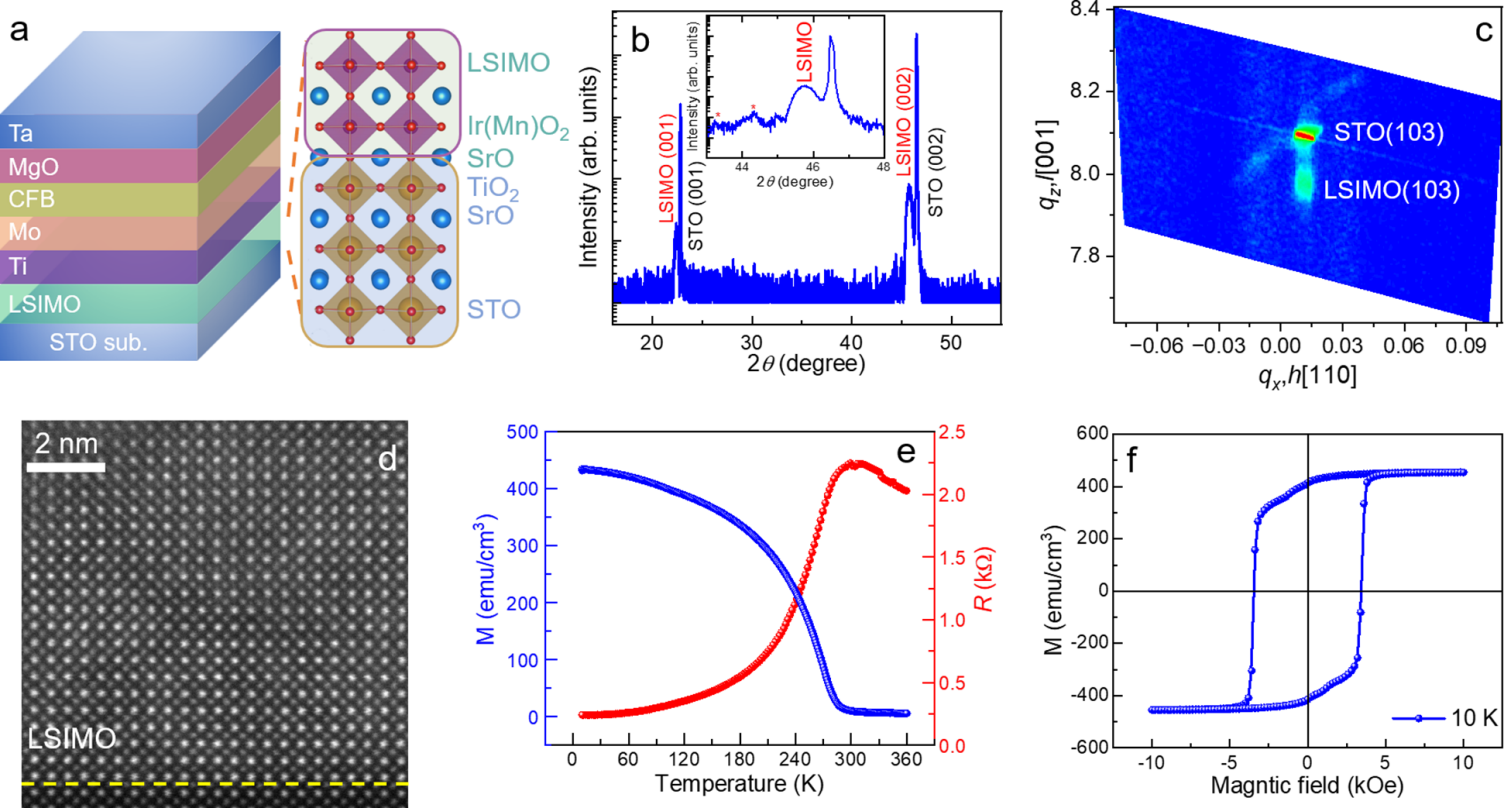
**Structural and magnetic properties characterization**. (**a**) Schematic of LSIMO/Ti/Mo/CFB/MgO/Ta multilayers and LSIMO growth on TiO_2_-terminated STO substrates. (**b**) XRD θ–2θ scan of the LSIMO/Ti/Mo/CFB/MgO/Ta multilayers with inset showing the (002) diffraction peak. (**c**) X-ray reciprocal space mapping around (103) peak of the LSIMO layer. (**d**) Cross-sectional STEM images of the LSIMO layer grown on the STO substrat**e**s. (**e**) Temperature-dependent magnetization and resistance of LSIMO layer, the sample was cooled from 360 K to 10 with an in-plane magnetic field of 1000 Oe, and then the magnetization signal was collected with an in-plane magnetic of 200 Oe during the warming process with a temperature increasing rate of 3 K/min. (**f**) *M–H* loop of the LSIMO layer measured at 10 K.

After confirming the high-quality epitaxial growth of the LSIMO layer, we investigated its magnetic and electrical transport properties. As shown in Figure 2e, the resistance of the LSIMO layer exhibits interesting behavior: it initially increases and then decreases monotonically with decreasing temperature, reaching a maximum around 290 K, which corresponds to the metal–insulator transition temperature.^51,52^ Above 290 K, the LSIMO layer shows semiconducting conductivity behavior, while below this temperature, it behaves metallically. The electric transport properties of the LSIMO layer are found to be sensitive to the growth temperature and the ratio of the LSMO and SIO (Figure S4). Similarly, the magnetization of the LSIMO film decreases with an increase in temperature, reaching zero around 290 K, which corresponds to the ferromagnetic Curie temperature of the LSIMO film. Figure 2f illustrates the in-plane *M-H* loop of the LSIMO layer at 10 K, showing a coercive field of approximately 3.5 kOe. This value is significantly higher than the approximately 60 Oe coercive field observed in LSMO thin film prepared under the same conditions (Figure S5). The enhanced coercive field of the LSIMO film is attributed to the strong SOC of iridium, which enhances the magnetic anisotropy of the LSIMO layers.^35,46−48^ Furthermore, Figure S6 presents the temperature-dependent *M-H* loops of the LSIMO layer, indicating that the coercive field decreases with increasing temperature.

The crystal structure characterization confirms the good crystallinity of the LSIMO films, while the magnetic and electrical characterizations reveal that the LSIMO layer behaves as a ferromagnetic conductor with strong SOC, suggesting the potential for generating *y*- and *z*-polarized spin currents through the spin Hall effect and SOPE. We then performed the current-induced magnetization switching in the LSIMO/Ti/Mo/CFB/MgO/Ta multilayers. The schematic of the device setup for measuring the SOT-driven magnetization switching is shown in Figure 3a. To evaluate the perpendicular magnetic anisotropy (PMA), the anomalous Hall resistance (*R*_H_) is measured with a small DC current of 100 μA applied along the *x*-axis and a magnetic field perpendicular to the Hall bar device. The rectangle-like hysteresis loop confirms the presence of PMA in the LSIMO/Ti/Mo/CFB/MgO/Ta multilayers (Figure 3b). Furthermore, as shown in Figure 3c, when a pulse current of 7.5 mA with a width of 300 μs is applied, the anomalous Hall effect (AHE) loop shifts toward the positive magnetic field direction, while it shifts toward the negative magnetic field direction when a pulse current of −7.5 mA is applied. This observed shift further confirms the existence of the out-of-plane damping like torque, which can support the field-free magnetization switching.^53−55^ It is worth mentioning that the AHE loop measured by applying a large pulse current exhibits a smaller coercive field of approximately 226 Oe, about 15% smaller than the value of approximately 266 Oe measured by a small DC current.

**Figure 3:**
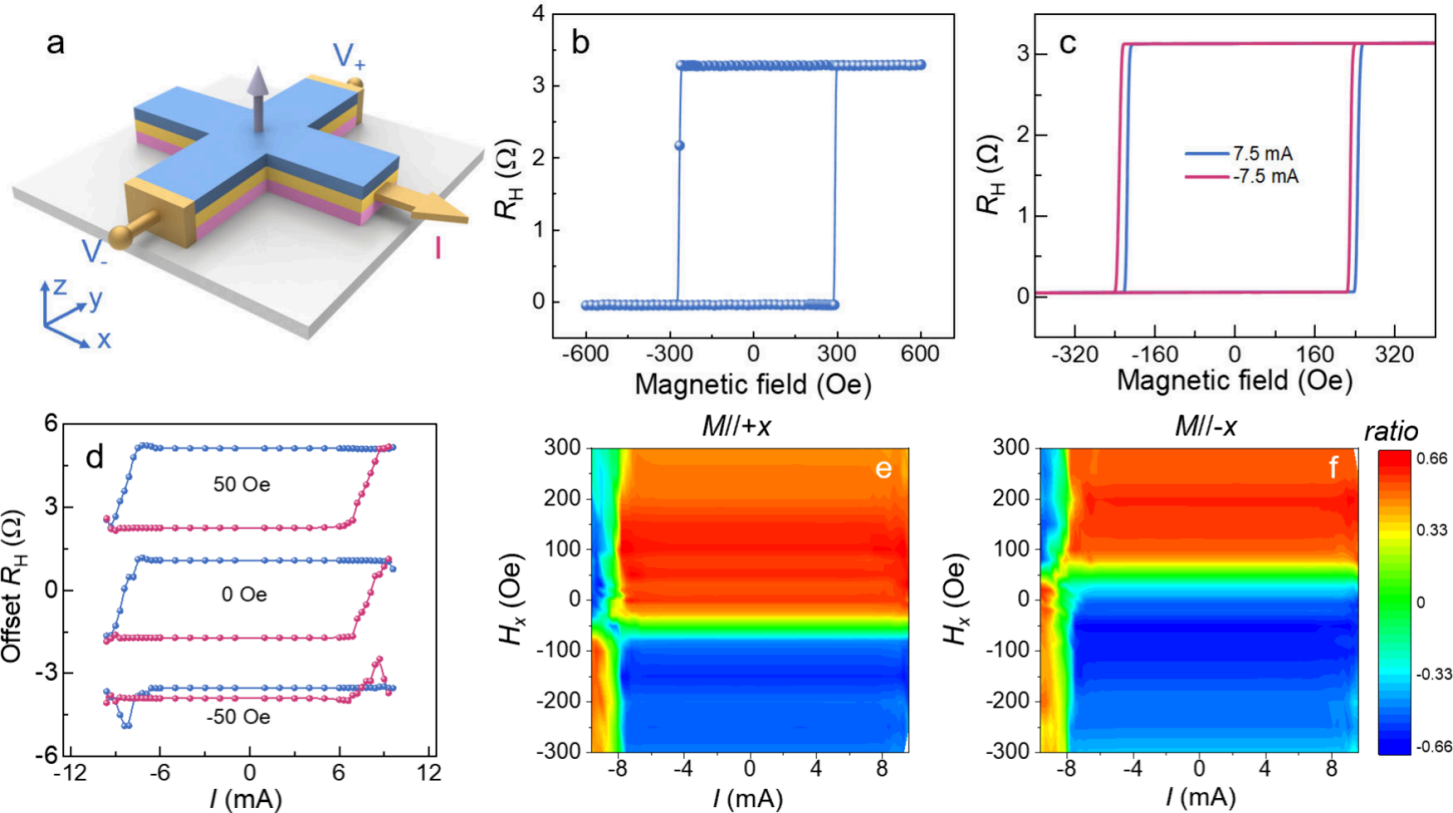
**Field-free switching of perpendicular magnetization**. (**a**) Schematic of the magnetization switching measurement setup using 300 μs current pulses. (**b**) Anomalous Hall effect of the LSIMO/Ti/Mo/CFB/MgO/Ta multilayers measured at 150 K. (**c**) Anomalous Hall loops measured with DC pulses of ±7.5 mA and 300 μs width. (**d**) Current-induced magnetization switching with an in-plane magnetic field *H*_*x*_ of 50, 0, and −50 Oe. The blue (red) curves represent the measured *R*_H_ when the pulse current is swept from the positive (negative) to negative (positive) direction. (**e**, **f**) Phase diagrams of switching ratio *r* verse current and magnetic field for LSIMO magnetized to +*x* (**e**) and −*x* (**f**) directions. The switching ratio *r* is defined as the switching amplitude divided by the anomalous Hall resistance.

We then performed the current-induced magnetization switching experiment at 150 K. Prior to the switching experiment, the in-plane magnetization of the LSIMO layer was premagnetized to the +*x* direction using an in-plane magnetic field of 1 T. The anomalous Hall resistance *R*_H_, which is proportional to the *z*-component of the magnetization (*M*_*z*_), is utilized to quantify the magnetization switching. The observations are summarized as follows (Figure 3d): First, the magnetization is successfully switched by both positive and negative pulse currents with different external magnetic fields applied along the *x*-axis. Second, current-induced magnetization switching at zero external magnetic field is also realized, indicating the limitation of nondeterministic switching of *y*-polarized spin current has been lifted. Third, the switching polarity remains unchanged under in-plane external magnetic fields of −50 and 50 Oe applied along the current direction. In conventional SOT systems, deterministic switching of perpendicular magnetization is unattainable as the perpendicular magnetization relaxes to either the +*z* or −*z* direction after the removal of the charge current (see Figure S8 in Supporting Information).^2−4^ Therefore, an in-plane magnetic field applied along the current direction is required to break the symmetry and achieve deterministic switching. For instance, when the magnetic field *H*_*x*_ is applied in the +*x* direction and the in-plane spin polarization (σ_*y*_) points in the −*y* direction (+*H*_*x*_, −σ_*y*_), the effective torque τ_*H*_ ∝ (σ_*y*_ × *H*_*x*_) acts in the −*z* direction. This torque aids the switching of magnetization from the +*z* to −*z* direction. Conversely, when the magnetic field is reversed, while the applied charge current remains unchanged, the direction of the effective torque changes from the −*z* to +*z* direction, favoring from −*z* to +*z* switching. The unchanged switching polarity and perpendicular magnetization switching at zero *H*_*x*_ indicate that the *z*-polarized spin current generated at the ferromagnetic LSIMO with a strong SOC is the probable origin.

The field-free switching of perpendicular magnetization is further found to depend strongly on the direction of the in-plane magnetization of the LSIMO layer. As shown in Figure 3e,f, we conducted a mapping measurement of the switching ratio *r* as a function of the current density *J* and the external field *H*_*x*_. The switching ratios are represented as negative (blue), zero (green), and positive (red), revealing two key observations. First, the critical magnetic field at which the switching polarity changes is influenced by the direction of the in-plane magnetization of the LSIMO layer. When the in-plane magnetization is premagnetized in the +*x* direction, the critical magnetic field is approximately −50 Oe. In contrast, when the in-plane magnetization is switched to the −*x* direction, the critical magnetic field shifts to around 50 Oe. Second, field-free magnetization switching occurs regardless of whether the magnetization is along the +*x* or −*x* directions. However, the direction of in-plane magnetization strongly influences the switching polarities at a zero magnetic field. Specifically, the switching polarity is anticlockwise when the in-plane magnetization is along the +*x* direction, as depicted in Figure 3d,e. This switching polarity changes to a clockwise position when the in-plane magnetization is switched to the −*x* direction. The in-plane magnetization-dependent field-free switching suggests that the switching of perpendicular magnetization in the CFB layer is correlated with the *z*-polarized spin current generated in the LSIMO layer with strong SOC and FM order.^21,33,49,50,56,57^ The damping-like torque τ_ADL,*y*_ = *m* × (σ_*y*_ × *m*) generated by the *y*-polarized spin current via the spin Hall effect dominates the entire switching process but is insufficient for deterministic magnetization switching alone. However, the out-of-plane damping-like torque τ_ADL,*z*_ = *m* × (σ_*z*_ × *m*) generated by the *z*-polarized spin current through the SOPE overcomes the limitations of *y*-polarized spin current and plays a crucial role in achieving deterministic perpendicular magnetization switching (see Figure S8).

The artificial LSIMO layer that exhibits both strong spin–orbit coupling (SOC) and ferromagnetic (FM) order should generate a total spin current with both the *z*-polarized spin current in addition to the *y*-polarized spin current due to the interaction between spin and magnetic order at the interface,^21,49,50,57−59^ which is validified by the second harmonic Hall voltage measurement result shown in Figure S7. The microscopic mechanism responsible for generating this *z*-polarized spin current arises from the precession of the spin current around the magnetization. From a phenomenological perspective, the source of this *z*-polarized spin current can be described by the following expression:
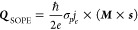
1where σ_*p*_ is the spin/charge conversion efficiency for the SOPE, ***s*** ≡ ***z*** × ***E***, ***Q***_SOPE_ is the out-of-plane spin current density generated by the SOPE, ***j***_*e*_ is the in-plane charge current density, ℏ is the reduced Planck’s constant, and *e* is the elementary electron charge.^21,49,50,57^ The generated ***Q***_SOPE_ depends on the angular relationship between ***M*** and ***s***. As shown in Figure 4a, the ***Q***_SOPE_ is proportional to the area of the orange region in the figure. It reaches its maximum when ***M*** is aligned along the *x*-axis and diminishes to zero when ***M*** is aligned along the *y*-axis.

**Figure 4:**
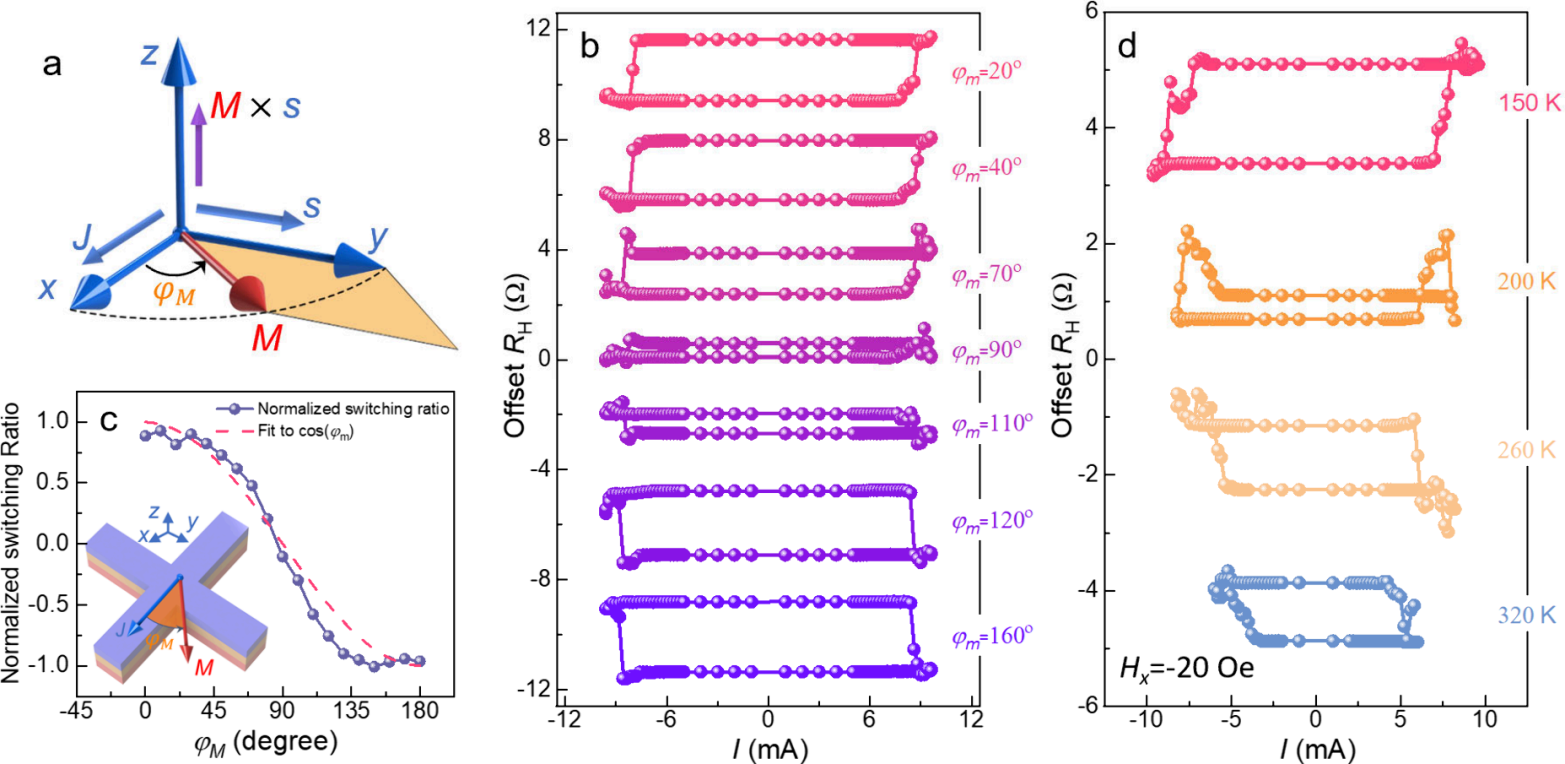
**Origin of the field-free magnetization switching**. (**a**) Schematic illustration of the *z*-polarized spin current generated by the SOPE effect. ***M*** represents the in-plane magnetization of the LSIMO layer with ***M*** × ***s*** being proportional to the area of the orange region in the diagram. (**b**) Current-induced magnetization switching measurement of the CFB layer, performed at different angles (φ_*M*_) between the ***M*** and the current flow direction. (**c**) Angular dependence of normalized switching ratio as a function of φ_*M*_. The inset shows a schematic of the measurement setup. (**d**) Current-induced magnetization switching at various temperatures, with a *H*_*x*_ = −20 Oe applied during the measurements.

To validate that the *z*-polarized spin current generated by the SOPE is the driving force behind field-free magnetization switching, we performed the angular-dependent magnetization switching measurement. Figure 4b shows the angular-dependent current-induced magnetization switching at *H*_*x*_ = 0. The measurement setup is schematically shown in the inset of Figure 4c. Before each measurement, the in-plane magnetization of the LSIMO layer is premagnetized by a 1 T magnetic field. The switching polarity is found to be angular-dependent. It shows anticlockwise switching polarity for φ_*M*_ < 90°, nearly vanished at φ_*M*_ = 90° (indicating the absence of *z*-polarized spin current), and then reversed to clockwise for φ_*M*_ > 90°. Additionally, the extracted switching ratio is angular-dependent and can be approximately described by cos φ_*M*_. This observation aligns with the behavior expected for the *z*-polarized spin current generated by SOPE, following the form ***Q***_SOPE_ ∝ (***M*** × ***s***). Thus, the magnetization switching polarity can be modulated by controlling the in-plane magnetization direction.

The generation of spin current with controllable spin polarization, modulated by the in-plane magnetization of the LSIMO layer, is further validated through temperature-dependent current-induced magnetization switching measurements. As shown in Figure 2e and Figure S6, the magnetic properties of the LSIMO layer exhibit clear temperature dependence, with both the saturation magnetization and coercive field decreasing as the temperature increases. According to Equation 1, the strength of the *z*-polarized spin current depends on both the magnitude and the orientation of the in-plane magnetization ***M*** in the LSIMO layer. Therefore, as the in-plane magnetization decreases with an increase in temperature, the *z*-polarized spin current decreases and finally vanishes around the Curie temperature of the LSIMO layer. This is confirmed by temperature-dependent SOT measurements. For these measurements, the sample is initially magnetized with a 1 T magnetic field, and a *H*_*x*_ of −20 Oe is applied during the SOT measurement. The change of switching polarity is found to be temperature-dependent: it is clockwise at 320 K but reverses to anticlockwise at 150 K. At 320 K, the LSIMO layer is in a paramagnetic state, where the absence of ferromagnetism prevents the generation of a *z*-polarized spin current. As a result, field-free magnetization switching does not occur, and the switching polarity remains clockwise under *H*_*x*_ = −20 Oe. When the temperature is decreased to 150 K well-below its Curie temperature, the LSIMO layer becomes ferromagnetic with a large coercive field (∼2300 Oe) and large saturation magnetization. In this state, a robust *z*-polarized spin current is generated, facilitating field-free magnetization switching. As a result, the switching polarity changes to anticlockwise, even with *H*_*x*_ = −20 Oe applied.

In conclusion, through interfacial reconstruction, an artificial ferromagnetic oxide thin-film LSIMO with strong SOC has been successfully prepared. The applied charge current can generate the *y*-polarized and *z*-polarized spin current through the spin Hall effect and SOPE. The *y*-polarized spin current dominates the perpendicular magnetization switching while the *z*-polarized spin current plays a key role in breaking the reversal symmetry achieving a field-free switching of perpendicular magnetization. Furthermore, the *z*-polarized spin current was found to be controllable by changing the direction of the in-plane magnetization of the LSIMO layer. Our findings proposed a new design by integrating SOC and ferromagnetic order together, which provides a new pathway for SOT-based magnetic random access memory technologies.

## Supporting Information Available

Discussion of the field-free switching. Experimental methods; Energy-dispersive X-ray spectroscopy of the LSIMO layer, atomic force microscopy characterization of LSIMO layer, temperature dependent resistance of the LSIMO layers, Magnetic properties of the LSIMO and LSMO single layers (PDF)
