# Supplementary material for: Controllable z-Polarized Spin Current in Artificially Structured Ferromagnetic Oxide with Strong Spin–Orbit Coupling
Source: Nano Lett. 2025 Jan 13;25(4):1528–35. doi: 10.1021/acs.nanolett.4c05502 (PMC11783597; doi:10.1021/acs.nanolett.4c05502)
Supplement: Supplementary file 1 — nl4c05502_si_001.pdf [file nl4c05502_si_001.pdf]

Supporting Information

Controllable *z*-polarized spin current in artificially structured ferromagnetic oxide with strong spin-orbit-coupling

Dongxing Zheng[1], Jingkai Xu[1], Qingxiao Wang[2], Chen Liu[1], Tao Yang[1], Aitian Chen[1], Yan Li[1], Meng Tang[1], Maolin Chen[1], Hanin Algaidi[1], Chao Jin[3], Kai Liu[4], Mathias Kläui[5], Udo Schwingenschlögl[1], and Xixiang Zhang[1*]

[1]Physical Science and Engineering Division, King Abdullah University of Science and Technology (KAUST), Thuwal 23955–6900, Saudi Arabia

[2]Corelab, King Abdullah University of Science and Technology (KAUST), Thuwal 23955–6900, Saudi Arabia

[3]Tianjin Key Laboratory of Low Dimensional Materials Physics and Processing Technology, School of Science, Tianjin University, Tianjin 300350, China.

[4]Physics Department, Georgetown University, Washington, DC 20057, USA

[5]Institute of Physics, Johannes Gutenberg University Mainz, 55099, Mainz, Germany

[*]Corresponding authors:

Xixiang Zhang (email: xixiang.zhang@kaust.edu.sa)

This PDF file includes the following:

Materials and Methods
Supplementary Text
Figs. S1 to S8
References

Materials and Methods

Sample growth

The $[(La_{0.67}Sr_{0.33}MnO_3)_{0.3}/(SrIrO_3)_{0.2}]_{120}$ (LSIMO) films with 120 periods were deposited on the TiO_2-terminated (001)-oriented $SrTiO_3$ (STO) substrates by a two-target deposition process using the $La_{0.67}Sr_{0.33}MnO_3$ (LSMO) and $SrIrO_3$ (SIO) targets. In each period, 0.3 unit cell (u.c.) LSMO and 0.2 u.c. SIO were deposited. LSMO target was fabricated using the conventional solid-state reaction method. Commercial $La_{0.67}Sr_{0.33}MnO_3$ powder (Sigma-Aldrich) was ground for two hours to achieve a finely ground and homogeneous consistency. Subsequently, the powder was mixed with ethanol before being pressed into a pellet with a diameter of 2 inches. The targets were then heated to 500 °C at a rate of 1 °C/min and maintained at this temperature for 6 hours to stabilize the shape and size. Following this, the temperature was increased to 1000 °C at the same rate, and the targets were sintered at this temperature for 12 hours before being cooled down to room temperature at 1°C/min. To achieve the TiO_2-terminated STO substrates, the STO substrates were first placed in boiling water for 10 min and then annealed in a tube furnace at 1000 °C in an oxygen partial pressure of 60 Torr for 6 h. The high quality of the surface of the substrates was confirmed through surface morphology measurements using atomic force microscopy.

The LSIMO films was fabricated by using the PLD deposition method. The growth rate of the LSMO and SIO were acquired by using the Reflection High Energy Electron Diffraction technique first. During the deposition, LSMO and SIO targets were ablated by a KrF excimer laser (wavelength = 248 nm) at a repetition rate of 3 Hz and a fluence of 1.35 J/cm^2. The substrate temperature was kept at 800 °C, and the oxygen partial pressure was maintained at 8 Pa. After the deposition, samples were annealed *in-situ* for 30 min at 800 °C in an oxygen pressure of 133 Pa. Finally, the samples were cooled to room temperature at a rate of approximately 10 °C/min in the same oxygen pressure of 133 Pa.

The Ti(3 nm)/Mo(2 nm)/CFB(0.8 nm)/MgO(2.2 nm)/Ta(2 nm) multilayers were deposited on the LSIMO layer by using a Singulus ROTARIS magnetron sputtering

system at room temperature with a base pressure of 1 × 10^{-6} Pa. After the deposition, the samples were annealed in a vacuum condition at 300 °C for 0.5 h with a perpendicular magnetic field of 8 kOe to get the perpendicular magnetic anisotropy.

Scanning transmission electron microscopy characterization

Cross-sectional TEM specimens were prepared using FIB milling in an FEI Helios 450 dual-beam system. High-angle annular dark-field (HAADF) scanning transmission electron microscopy (STEM) imaging and energy-dispersive X-ray (EDX)/electron energy loss spectroscopy (EELS) mapping were carried out at 300 kV in an FEI Titan Themis Z microscope equipped with in-column (Super-X) EDX detectors, a high brightness field emission gun (XFEG), a spherical aberration (CS) corrector for the probe forming system.

Electrical transport characterization

Conventional photolithography, electron beam lithography, and ion-milling techniques were used to pattern the heterostructures into Hall bars. The width of the Hall bar is 3 μm. The Ti/Au with a thickness of 10/100 nm, respectively, deposited by a magnetron sputtering method was used as electrodes. The anomalous Hall effect, harmonic Hall resistance, and current-induced magnetization switching were measured using Hall bar devices. The electrical transport properties and magnetic properties were measured using a Quantum Design Physical Property Measurement System (PPMS) and Quantum Design DynaCool system. The first and second harmonic Hall resistances (voltages) were measured using two Stanford SR830 lock-in amplifiers. The Keithley 6221/2182A Source Meter/Nanovoltmeter combination which can generate the pulse current with a pulse width from 50 μs to 12 ms was used to generate the current pulse with the pulse width of 300 μs. Another two Keithley 6221 Source Meter and 2182A Nanovoltmeter were used to measure the Hall voltage.

Supplementary Text

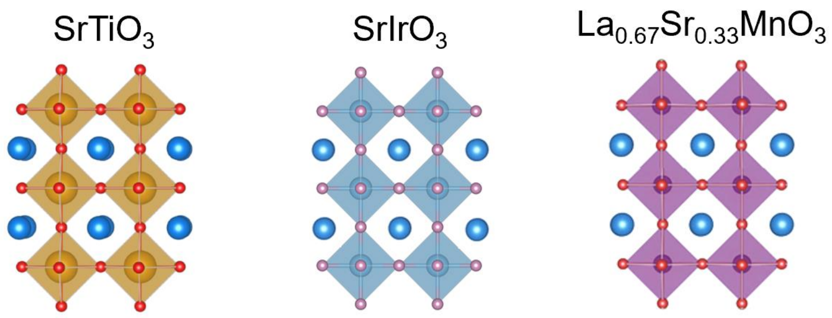

FIG. S1. Schematics of the crystal structures of $SrTiO_3$, $La_{0.67}Sr_{0.33}MnO_3$, and $SrIrO_3$.

The lattice parameters of the $SrTiO_3$ substrate with cubic perovskite structure is 3.905 Å, making it an ideal substrate for the epitaxial growth of $La_{0.67}Sr_{0.33}MnO_3$, and $SrIrO_3$ layers due to the relatively small lattice mismatch and similar crystal structures. SIO exhibits a distorted perovskite structure in the centrosymmetric Pbnm space group, with lattice constants a_0=5.60 Å, b_0=5.58 Å, and c_0=7.75 Å, which corresponds to a pseudocubic lattice constant $a=\sqrt{\left(2a_0^2+2b_0^2+c_0^2\right)/12}=3.93$ Å.[1] The crystal structure of bulk LSMO is rhombohedral with a pseudocubic lattice parameter of a=3.87 Å.

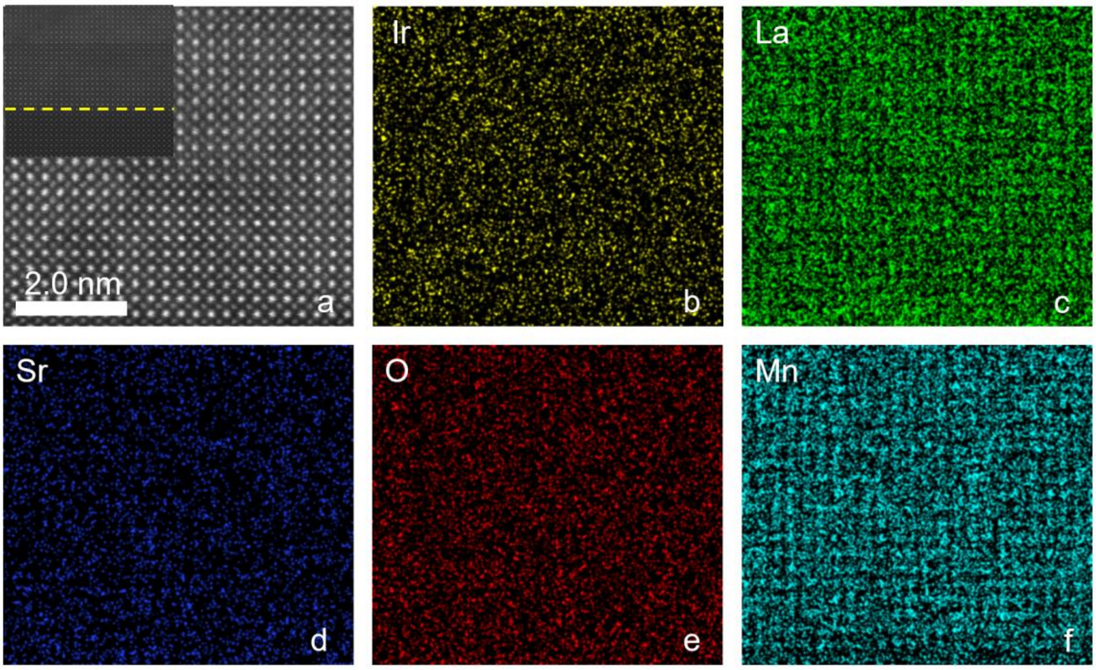

FIG. S2. STEM cross-section image of the LSIMO film. The EDXS mapping of Ir, La, Sr, O and Mn were collected in the same area where the STEM image was taken.

Figure S2 presents an atomic resolution scanning transmission electron microscopy (STEM) image of the LSIMO film, showcasing its well-ordered atomic arrangement and indicating high-quality growth. Inset of Fig. S2a shows a broader view of the LSIMO/STO substrates, highlighting clear contrast at the interface, due to the difference in the atomic z number of each element. The distinct and well-defined interface between layers is evident. Additionally, energy-dispersive X-ray spectroscopy (EDXS) mapping of Ir, La, Sr, O, and Mn elements demonstrates uniform atom distribution within the LSIMO films, further corroborating their high-quality growth.

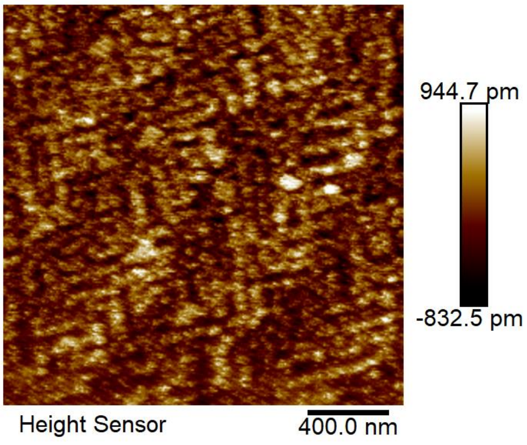

FIG. S3. Surface morphology of the LSIMO film.

Fig. S3 shows the surface morphology of the LSIMO film. The surface is flat with the surface fluctuation in ± 1 nm.

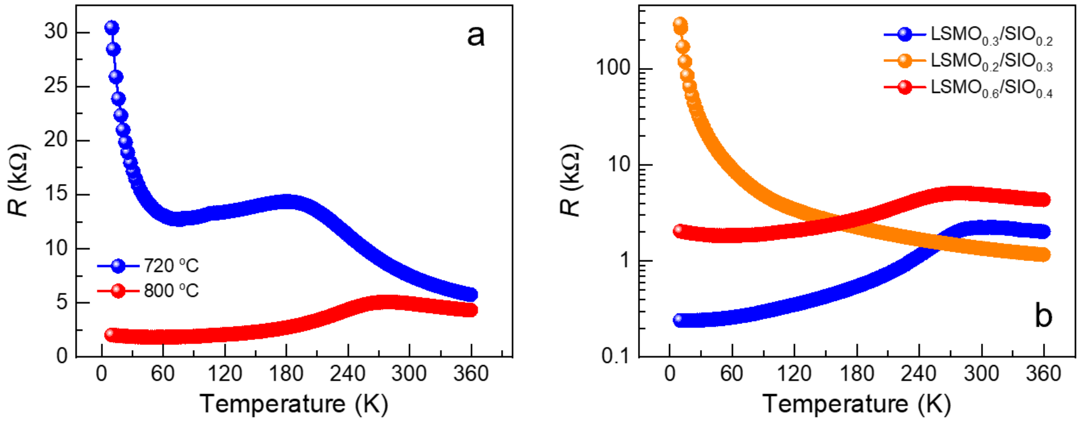

FIG. S4. Temperature dependent resistance of the LSMO-SIO films grown at different conditions. **a** *R-T* curves of the 24 nm thick $LSMO_{0.6}/SIO_{0.4}$ films grown at different temperatures. **b** *R-T* curves of the LSIMO with different ratios of the LSMO and SIO.

The electrical transport properties of the LSIMO films are found to be influenced by the growth temperature and the ratio of LSMO to SIO. As shown in Fig. S4a, the 24 nm thick $LSMO_{0.6}/SIO_{0.4}$ films grown at 800 and 720°C exhibit completely different conducting behaviors. The resistance of the 24 nm thick $LSMO_{0.6}/SIO_{0.4}$ film grown at 800°C increases with decreasing temperature, reaching a maximum around 270 K, and

then decreases with further temperature decrease. In contrast, for the $LSMO_{0.6}/SIO_{0.4}$ film grown at 720°C, the resistance increases rapidly at low temperatures. The LSMO/SIO ratio was found to play a significant role in influencing the electrical transport of the LSMO-SIO films. As shown in Fig. S4b, the $LSMO_{0.3}/SIO_{0.2}$ and $LSMO_{0.6}/SIO_{0.4}$ films show a semiconductor-to-metallic transition around 290 K and 270 K, respectively, while the $LSMO_{0.2}/SIO_{0.3}$ film exhibits semiconducting conductive behavior.

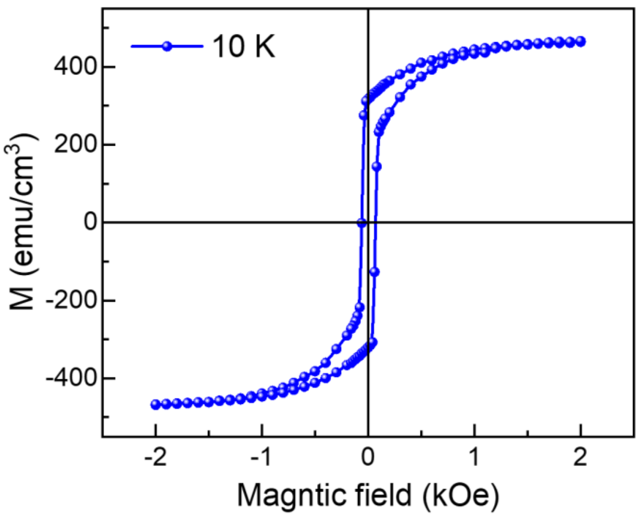

FIG. S5. *M-H* loop of the LSMO single layer at 10 K.

Figure S5 shows the *M-H* loop of the LSMO single layer fabricated under the same conditions as the LSIMO layer. In contrast to the LSIMO layer's high coercive field of 3500 Oe at 10 K, the LSMO single layer exhibits a much lower coercive field of 63 Oe, indicative of typical soft magnetic behavior.

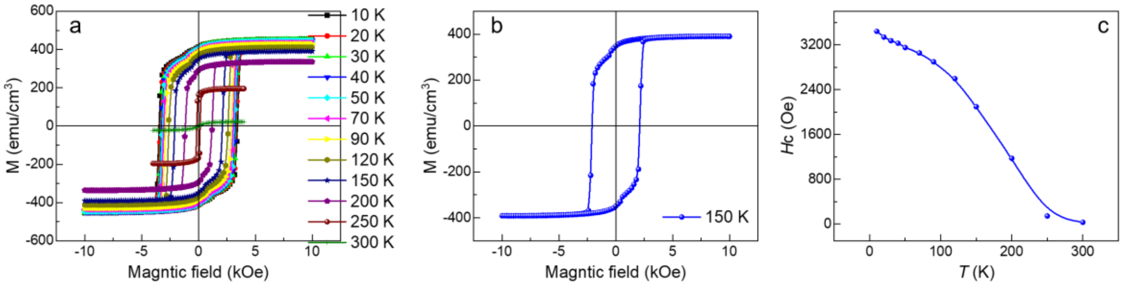

FIG. S6. **a** *M-H* loop of LSIMO layer at different temperatures. **b** *M-H* loop of LSIMO layer at 150 K. **c** Temperature dependent coercive field of the LSIMO layer.

Figure S6a presents the *M-H* loop of the LSIMO single layer at different temperatures, revealing a square-shaped loop below 300 K, indicative of coherent magnetization rotation at low temperatures. With increasing temperature, both saturation magnetization and coercive field decrease. The *M-H* loop of the LSIMO layer at 150 K (Fig. S6b) displays a saturation magnetization of approximately 400 emu/cm^3 and a coercive field of around 2100 Oe, suggesting hard magnet behavior, in contrast to the soft magnetic behavior observed in the single LSMO layer (Fig. S4). Additionally, Fig. S6c illustrates the temperature-dependent coercive field of the LSIMO layer, showing a decrease from approximately 3500 Oe at 10 K to about 30 Oe at 300 K.

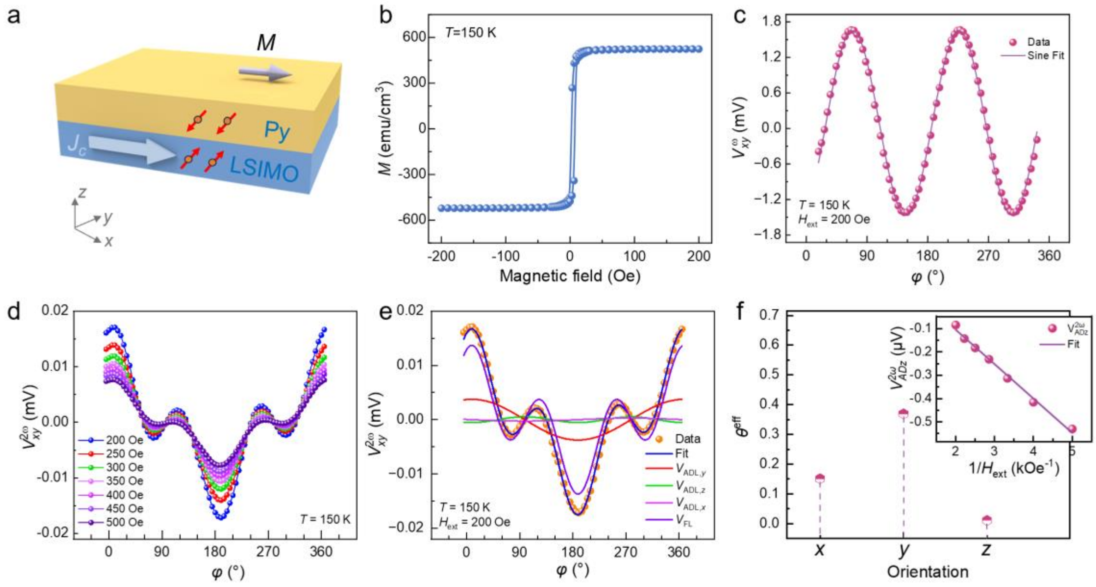

FIG. S7. **a** Schematic diagram of the second harmonic Hall voltage measurement setup. **b** *M*-*H* loop of the Py layer measured with an in-plane magnetic field at 150 K. **c** V_{xy}^{w} as a function of a rotating in-plane magnetic field (200 Oe) of the LSIMO/Ti/Mo/CFB/MgO/Ta multilayers at 150 K. **d** Second-harmonic Hall voltage $V_{xy}^{2\omega}$ as a function rotating in-plane magnetic fields. **e** Second-harmonic Hall voltage $V_{xy}^{2\omega}$ as a function of in-plane external magnetic field of 200 Oe for the multilayers at 150 K, where the blue curve is the fit to Eq. (1). Both cosφ (red curve), cos2φ (light green curve), sinφ (pink curve) and cos2φsinφ (purple curve) are revealed. **f** Calculated effective spin Hall angle of *x*-, *y*- and *z*-polarized spin current. The inset shows the 1/H_{ext} dependence of the fitting parameter $V_{\text{AD},z}^{2\omega}$.

We conducted second harmonic Hall voltage (SHH) measurements on the LSIMO/Ru(3 nm)/Py(4 nm)/Ru(3 nm) multilayers. During the SHH measurement, an AC current $i = I\sin(\omega t)$ is applied to the Hall bar. The induced effective spin-orbit

fields oscillate the magnetization around its equilibrium position, causing the Hall resistance R(t) to oscillate at the same frequency ω. Consequently, the Hall voltage $V(t) = R(t)I\sin(\omega t)$ measured at 2ω frequency contains a second harmonic component directly related to the current-induced fields.[2, 3] The sample is rotated in the *x*-*y* plane with the applied in-plane magnetic fields significantly larger than the saturation magnetic field, maintaining the magnetization in a single domain state. The ferromagnetic LSIMO with strong SOC is expected to generate *y*-, *x*-, and *z*-polarized spin currents through the spin Hall effect, spin anomalous Hall effect, and spin-orbit precession effect. Consequently, accumulated spin currents exert torques $\tau_{\mathrm{ADL},x}$, $\tau_{\mathrm{ADL},y}$ and $\tau_{\mathrm{ADL},x}$, τ_{FL} on the Py layer along the in-plane and out-of-plane directions, respectively.

Figure S7b shows the *M*-*H* loop of the Py layer at 150 K, revealing a hysteresis loop with a saturation magnetic field as low as 10 Oe. This value is significantly lower than the applied minimum external magnetic field of 200 Oe, indicating that the Py layer can be fully magnetized during the SHH measurement. Fig. S7c shows the harmonic Hall voltage V_{H}^{ω} as a function of the azimuthal angle φ, measured under an external magnetic field of 200 Oe at 150 K. The variation of V_{H}^{ω} fits well with sin2φ, indicating consistent alignment of the magnetization of Py layer with the direction of the external in-plane magnetic field. Fig. S7d shows the second harmonic Hall voltage $V_{\mathrm{H}}^{2\omega}$ as a function of the azimuthal angle φ, measured with the magnetic field ranges from 200 to 500 Oe at 150 K. Figure R7e shows $V_{\mathrm{H}}^{2\omega}$ as a function of φ, measured

under the same magnetic field and temperature. The second harmonic Hall voltages can be described by the following equations.[2, 4-8]

$$V_{2\omega} = -V_{\mathrm{ADL},y}\cos(\varphi+\varphi_0) + V_{\mathrm{FL}} cos2(\varphi+\varphi_0)\sin(\varphi+\varphi_0) + V_{\mathrm{ADL},z} cos2(\varphi+\varphi_0) - V_{\mathrm{ADL},x}\sin(\varphi+\varphi_0) + V_{\mathrm{PNE}}\, sin2(\varphi+\varphi_0) + V_{2\omega 0}, \quad (1)$$

where

$$V_{\mathrm{ADL},y} = \frac{1}{2} V_{\mathrm{AHE}} \frac{H_{\mathrm{ADL},y}}{H_x+H_k} + V_{\mathrm{ANE}}, \quad (2)$$

$$V_{\mathrm{ADL},x} = \frac{1}{2} V_{\mathrm{AHE}} \frac{H_{\mathrm{ADL},x}}{H_x+H_k}, \quad (3)$$

$$V_{\mathrm{ADL},z} = V_{\mathrm{PHE}} \frac{H_{\mathrm{ADL},z}}{H_x}, \quad (4)$$

$$V_{\mathrm{FL}} = V_{\mathrm{PHE}} \frac{H_{\mathrm{FL}}+H_{\mathrm{Oe}}}{H_x}. \quad (5)$$

Here, $V_{2\omega 0}$, φ_0, V_{AHE}, V_{ANE}, and V_{PHE} are device offset, angular offset, the anomalous Hall voltage, anomalous Nernst voltage, and planar Hall voltage, respectively. $H_{\mathrm{ADL},y}$, $H_{\mathrm{ADL},x}$, $H_{\mathrm{ADL},z}$, and H_{FL} are spin-orbit fields corresponding to the torque $\tau_{\mathrm{ADL},y}$, $\tau_{\mathrm{ADL},x}$, $\tau_{\mathrm{ADL},z}$, and τ_{ADL}, respectively.

Since the current-induced torques have different dependencies on the azimuthal angle φ, therefore, their contributions in the second harmonic Hall voltage can be extracted. Their contributions in the $V_{\mathrm{H}}^{2\omega}$, as indicated by the angular-dependent $V_{\mathrm{ADL},y}$ (purple line), $V_{\mathrm{ADL},z}$ (light line), $V_{\mathrm{ADL},x}$ (purple line), and V_{FL} (purple line) are shown in Fig. S7e. By fitting the extracted parameter at different external magnetic fields, we can obtain the correlations between $V_{\mathrm{ADL},x,y,z}$ and V_{FL} and external magnetic fields. According to Equations 1-5, slopes of linear fits to the $V_{\mathrm{ADL},x,y}$ as a function$1/(H_x+H_k)$ give the information about $H_{\mathrm{ADL},x,y}$, and $V_{\mathrm{ADL},z}$ as a function of $1/H_x$ give the information about $H_{\mathrm{ADL},z}$. The effective spin Hall angle of the *x*-, *y*- and

z- polarized spin current were calculated by using $\theta_{x,y,z}^{eff} = \frac{2eM_S t_{Py} \mu_0 H_{\mathrm{ADL},x,y,z}}{\hbar J}$, where e is the electron charge, $\hbar$ is the reduced Planck constant, M_S is the saturation magnetization of Py layer, and J is the applied current density. As is shown in Fig. S7f, the effective spin Hall angles of the x-, y-, and z-polarized spin currents are 0.151, 0.369, 0.011, respectively. The ratio of the out-of-plane (z-polarized) and in-plane (y-polarized) spin polarization is ~0.030. This value is slightly larger than the value of ~0.021 in the $MnPd_3$ based system, where a z-spin polarization assisted field-free magnetization switching has been achieved.[7]

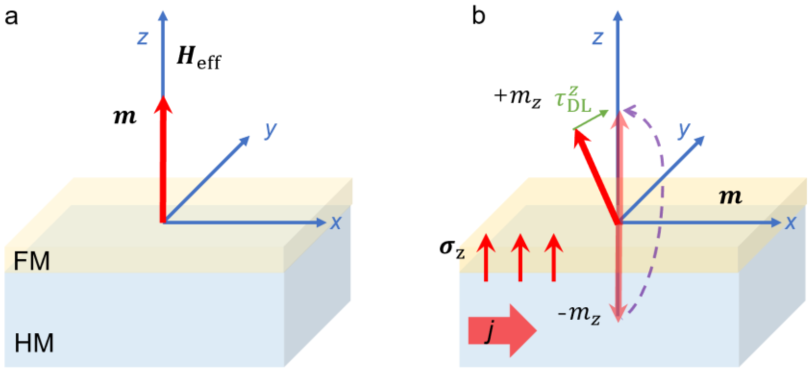

FIG. S8 Magnetization switching with a charge current along *x*-axis. **a** Before the application of charge current, **b** During the application of a large charge current.

As is shown in Fig. S8a, $\boldsymbol{H}_{\text{eff}}$ is the effective magnetic field of the CFB layer along the out-of-plane direction. When the magnetization $\boldsymbol{m}$ is away from the equilibrium, the magnetization $\boldsymbol{m}$ precesses around the local effective field and finally goes to the equilibrium position due to the energy dissipation during the precession. This process is governed by the Landau-Lifshitz-Gilbert (LLG) equation, which can be expressed as[9]:

$$\frac{\partial \boldsymbol{m}}{\partial \boldsymbol{t}} = \boldsymbol{\tau}_p + \boldsymbol{\tau}_{\boldsymbol{D}} = -\gamma \boldsymbol{m} \times \boldsymbol{H}_{\text{eff}} + \alpha \boldsymbol{m} \times \frac{\partial \boldsymbol{m}}{\partial t} \quad (6)$$

The first term is the field-like term generated by an effective field. The second term is the damping term, which will finally push the magnetization to the equilibrium position. γ is the gyromagnetic ratio and α is the Gilbert damping parameter.

When a sufficiently large charge current is applied on the *x* axis, a spin current with the spin polarization along the *y*-axis will be generated. At this time, the two terms of the damping-like and field-like torques should also be included in the LLG equation:

$$\frac{\partial \boldsymbol{m}}{\partial t} = -\boldsymbol{\gamma} \boldsymbol{m} \times \boldsymbol{H}_{\text{eff}} + \boldsymbol{\alpha} \boldsymbol{m} \times \frac{\partial \boldsymbol{m}}{\partial t} + \boldsymbol{\alpha}_J \boldsymbol{m} \times \boldsymbol{\sigma}_y + \boldsymbol{\alpha}_J \boldsymbol{m} \times (\boldsymbol{m} \times \boldsymbol{\sigma}_y) \quad (7)$$

The equilibrium position of $\boldsymbol{m}$ satisfies the condition $\frac{\partial \boldsymbol{m}}{\partial \boldsymbol{t}} = 0$. Here, it is obvious that equation (2) meets the requirement $\frac{\partial \boldsymbol{m}}{\partial t} = 0$ when $\boldsymbol{m}//\boldsymbol{y}$, where m_z vanishes and therefore the anisotropy field becomes zero. However, such a configuration is not the ground state of the macrospin. As is shown in Fig. S8b, the magnetization will symmetrically relax to either the $+m_z$ and $-m_z$ directions after the removal of charge current, leading to a non-deterministic magnetization switching.

Now, let's consider the case with *z*-polarized spin polarization. In the case with $\boldsymbol{\sigma}_z$, the magnetic dynamics of the magnetization $\boldsymbol{m}$ is governed by the LLG equation, which can be expressed as:

$$\frac{\partial \boldsymbol{m}}{\partial t} = -\gamma \boldsymbol{m} \times \boldsymbol{H}_k + \alpha \boldsymbol{m} \times \frac{\partial \boldsymbol{m}}{\partial t} - \alpha_J \boldsymbol{m} \times \boldsymbol{\sigma}_z - \alpha_J \boldsymbol{m} \times (\boldsymbol{m} \times \boldsymbol{\sigma}_z) \qquad (8)$$

The equilibrium position of $\boldsymbol{m}$ satisfies the condition $\frac{\partial \boldsymbol{m}}{\partial t} = 0$. It can be expected that $\frac{\partial \boldsymbol{m}}{\partial t} = 0$ when $\boldsymbol{m} // \boldsymbol{z}$, which indicates that the $\boldsymbol{\sigma}_z$ is able to switch the magnetization to the *z* direction. Here, it should be mentioned that both the +z and -z direction meets the requirement of $\frac{\partial \boldsymbol{m}}{\partial t} = 0$. However, the +*z* direction is the ground state for the magnetization $\boldsymbol{m}$, while -*z* is the meta-stable position. When a spin current with the spin polarization of $+\boldsymbol{\sigma}_z$ acts on the magnetization $\boldsymbol{m}$, the magnetization along the -*z* direction will be switched to the +z due to a perturbation. Thus a deterministic switching of perpendicular magnetization $\boldsymbol{m}$ is allowed by using a spin current with *z*-spin polarization. In the case of $-\boldsymbol{\sigma}_z$, we can rotation $\boldsymbol{m}$ from +*z* to -*z*.

REFERENCES

(1) Chamberland, B.; Philpotts, A. Chemical constitution of various SrIrO3 phases. *J. Alloys Compd.* **1992**, *182* (2), 355-364.
(2) Avci, C. O.; Garello, K.; Gabureac, M.; Ghosh, A.; Fuhrer, A.; Alvarado, S. F.; Gambardella, P. Interplay of spin-orbit torque and thermoelectric effects in ferromagnet/normal-metal bilayers. *Phys. Rev. B* **2014**, *90* (22), 224427. DOI: 10.1103/PhysRevB.90.224427.
(3) Pi, U. H.; Won Kim, K.; Bae, J. Y.; Lee, S. C.; Cho, Y. J.; Kim, K. S.; Seo, S. Tilting of the spin orientation induced by Rashba effect in ferromagnetic metal layer. *Appl. Phys. Lett.* **2010**, *97* (16), 162507. DOI: 10.1063/1.3502596.
(4) Wang, Y.; Zhu, D.; Yang, Y.; Lee, K.; Mishra, R.; Go, G.; Oh, S.-H.; Kim, D.-H.; Cai, K.; Liu, E. Magnetization switching by magnon-mediated spin torque through an antiferromagnetic insulator. *Science* **2019**, *366* (6469), 1125-1128.
(5) Shao, Q.; Tang, C.; Yu, G.; Navabi, A.; Wu, H.; He, C.; Li, J.; Upadhyaya, P.; Zhang, P.; Razavi, S. A.; et al. Role of dimensional crossover on spin-orbit torque efficiency in magnetic insulator thin films. *Nat. Commun.* **2018**, *9* (1), 3612. DOI: 10.1038/s41467-018-06059-7.
(6) Wen, Y.; Wu, J.; Li, P.; Zhang, Q.; Zhao, Y.; Manchon, A.; Xiao, J. Q.; Zhang, X. Temperature dependence of spin-orbit torques in Cu-Au alloys. *Phys. Rev. B* **2017**, *95* (10), 104403. DOI: 10.1103/PhysRevB.95.104403.
(7) Dc, M.; Shao, D. F.; Hou, V. D.; Vailionis, A.; Quarterman, P.; Habiboglu, A.; Venuti, M. B.; Xue, F.; Huang, Y. L.; Lee, C. M.; et al. Observation of anti-damping spin-orbit torques generated by in-plane and out-of-plane spin polarizations in MnPd(3). *Nat. Mater.* **2023**, *22* (5), 591-598. DOI: 10.1038/s41563-023-01522-3.
(8) Bose, A.; Schreiber, N. J.; Jain, R.; Shao, D.-F.; Nair, H. P.; Sun, J.; Zhang, X. S.; Muller, D. A.; Tsymbal, E. Y.; Schlom, D. G.; et al. Tilted spin current generated by the collinear antiferromagnet ruthenium dioxide. *Nat. Electron.* **2022**, *5* (5), 267-274. DOI: 10.1038/s41928-022-00744-8.
(9) Gilbert, T. L. Classics in Magnetics A Phenomenological Theory of Damping in Ferromagnetic Materials. *IEEE Trans. Magn.* **2004**, *40* (6), 3443-3449. DOI: 10.1109/tmag.2004.836740.
